# Stimuli-Responsive Nanodiamond–Polyelectrolyte Composite Films

**DOI:** 10.3390/polym12030507

**Published:** 2020-02-26

**Authors:** Tony Tiainen, Marina Lobanova, Erno Karjalainen, Heikki Tenhu, Sami Hietala

**Affiliations:** Department of Chemistry, University of Helsinki, PB 55, FIN-00014 Helsinki, Finland; tony.tiainen@helsinki.fi (T.T.); lobanovams8@gmail.com (M.L.); erno.karjalainen@helsinki.fi (E.K.); heikki.tenhu@helsinki.fi (H.T.)

**Keywords:** nanodiamond, polyelectrolyte, nanocomposite, nanofiller, PDMAEMA, LCST, thermoresponsive

## Abstract

Nanodiamonds (NDs) can considerably improve the mechanical and thermal properties of polymeric composites. However, the tendency of NDs to aggregate limits the potential of these non-toxic, mechanically- and chemically-robust nanofillers. In this work, tough, flexible, and stimuli-responsive polyelectrolyte films composed of cross-linked poly(butyl acrylate-*co*-dimethylaminoethyl methacrylate) (P(BA-*co*-DMAEMA)) were prepared by photopolymerization. The effects of the added carboxylate-functionalized NDs on their mechanical and stimuli-responsive properties were studied. When the negatively charged NDs were added to the polymerization media directly, the mechanical properties of the films changed only slightly, because of the uneven distribution of the aggregated NDs in the films. In order to disperse and distribute the NDs more evenly, a prepolymerized polycation block copolymer complexing agent was used during the photopolymerization process. This approach improved the mechanical properties of the films and enhanced their thermally-induced, reversible phase-transition behavior.

## 1. Introduction

In composites, nanofillers have an advantage over microfillers, because of their increased surface area and, consequently, higher interfacial adhesion, which enhances the mechanical properties of the composites [1,2,3,4,5]. Nanodiamonds (NDs) are ~5 nm sized carbon particles in which sp^3^ hybridized carbon is in a diamond cubic crystal structure, surrounded with sp^2^ carbon on the shell. NDs retain the many beneficial properties of a macrodiamond, down to the nanoscale. These properties range from mechanical hardness and a high Young’s modulus; thermal conductivity; and wear-, corrosion-, and chemical-resistance, to a good biocompatibility [6]. Furthermore, NDs have a rich and versatile surface chemistry [7,8,9,10,11], because of the accessible sp^2^ and sp^3^ carbons at the surface. Owing to the large surface-area-to-volume ratio of the nanoparticles, much of the ND surface is in contact with the surrounding environment, enhancing the interactions between the ND and its surroundings [12]. This, combined with the ND surface chemistry and diamond-like structure of the core, make NDs a potential nanofiller for composites.

The use of NDs as fillers of polymer composites has been studied for thermoplastics, thermosets, and biopolymer-based and elastomeric materials [6,11,12,13,14,15,16,17,18,19,20]. The studies show that the addition of NDs into these materials improves their mechanical properties. However, common problems are related to the uneven distribution of the NDs in the matrix and the aggregation of NDs. These lower their active surface area, causing the physical reinforcing effect to deteriorate [18]. Consequently, efficient dispersion is required through the manufacturing process, which can be achieved by choosing a proper ND surface to accommodate them with a specific matrix [21,22]. 

Polyelectrolyte films are important components, for example, in ion exchange [23], gas separation [24], and water purification [25] membranes. Poly(dimethylaminoethyl methacrylate) (PDMAEMA), a weak polycation, is an interesting material for these applications, especially as it has a pH, ionic strength, and temperature-responsiveness that can be utilized in advanced material applications. Correspondingly, DMAEMA containing copolymer films has been studied [26,27] and utilized as a CO_2_ separation membrane, exhibiting high permeability and gas selectivity [28,29], and as an anion exchange membrane with improved cation-gating properties [30]. The stimuli-responsivity of PDMAEMA has inspired studies of PDMAEMA membranes for their ability to variate the flux of water through them [31,32], their use as oil/water separating membranes with the capability of using temperature or pH as a trigger [33], and for actuation purposes [34]. Polyelectrolytes have been shown to also aid in the aggregation prevention and dispersion of NDs [35,36].

In order to increase the interfacial adhesion between the filler and the matrix, additional compatibilizers are often used [3]. The compatibilizer can be covalently bound to the fillers or rely on non-covalent interactions, such as Van der Waals- or electrostatic interactions. In the case of non-covalent compatibilizers, in particular, graft- and block-copolymers are useful as they can have interactions with the fillers and are simultaneously miscible with the matrix.

In this work, cross-linked poly(butyl acrylate-*co*-dimethylaminoethyl methacrylate) (P(BA-*co*-DMAEMA)) polyelectrolyte films containing varying amounts of NDs and polymer fillers were prepared by a simple photopolymerization method. The cationic DMAEMA units were expected to have interactions with the carboxylated NDs. First, the ND content was varied to study the effects of NDs on the properties of the films. Then, a block copolymer complexing agent, poly(dimethylaminoethyl methacrylate)-*block*-poly(ethylene oxide) (PDMAEMA-*b*-PEO), was used as a compatibilizer for the NDs. This complexing agent was expected to disperse and stabilize the NDs in the polymerization mixture during the film preparation, enhancing the incorporation of NDs into the film matrix. The mechanical and stimuli-responsive properties of the films were studied. 

## 2. Materials and Methods

### 2.1. Materials

The monomers, butyl acrylate (BA; 99%, Sigma-Aldrich, Helsinki, Finland) and *N*,*N*-dimethylaminoethyl methacrylate (DMAEMA; 99%, Acros Organics/VWR, Vantaa, Finland), as well as the cross-linker, 1,4-Butanediol dimethacrylate (BuDMA; 95%, Sigma-Aldrich, Helsinki, Finland), were distilled under reduced pressure, and were run through an anhydrous alumina column and a filter to remove the inhibitors and oligomers. The photoinitiator, 2-hydroxy-2-methylpropiophenone (97%, Sigma-Aldrich, Helsinki, Finland), carboxylated detonation nanodiamond powder (μDiamond® Molto Vox; Carbodeon Oy, Vantaa, Finland), and acetonitrile (ACN; 99.9%, Sigma-Aldrich, Helsinki, Finland) were used as received. Deionized ultrapure water with ~18 mΩ/cm resistivity was used as received from the purifier. Carbonate buffer (AVS Titrinorm; VWR, Helsinki, Finland) was used as received.

### 2.2. Block Copolymer Complexing Agent

The block copolymer complexing agent, PDMAEMA-*b*-PEO, was synthesized via reversible addition−fragmentation chain-transfer polymerization (RAFT) using a polyethylene oxide (PEO)-based chain transfer agent (CTA) (*M* 1100 g/mol) as reported earlier [36]. According to the gel permeation chromatography (GPC), the polymer had a *M*_n_ 15,000 g/mol and a dispersity of 1.6.

### 2.3. Film Preparation (Scheme 1)

The polymerization mixtures were prepared by weighing a predetermined ratio of the monomers BA and DMAEMA, and adding 2.5 wt % cross-linker BuDMA into a 20 mL glass vial and shaking until homogenous (Table 1 and Table S1). For the samples prepared with the complexing agent, the polymer PDMAEMA-*b*-PEO was added to the mixture with the monomers and the cross-linker, and the mixing was continued until the polymer was dissolved. In the case of the ND–polymer composite films, the NDs were added to the polymerization mixture and the mixture was sonicated for 15 min (Hielscher UP400S Ultrasonic Processor (Teltow, Germany) with an H3 sonotrode at 400 W, 0.5 cycle and an amplitude of 100%). The ND–polymer composites with the complexing agent were prepared by adding the NDs into the polymerization mixture with all of the other components, followed by sonication for 15 min. During sonication, the vial was kept in an ice bath (~0 °C) to prevent heating and polymerization. After mixing or mixing and sonication, the initiator was added and the prepared dispersion was placed on a Teflon coated Petri dish covered with a lid coated with semitransparent Teflon tape (Figure S8, Supplementary Materials). The mixtures were cured under four 365 nm 9 W UV-lights for 2 h, followed by overnight washing with acetonitrile to remove the excess monomers and the initiator. Washed films were dried under ambient conditions for 24 h, followed by a 2 h drying in a vacuum desiccator at 50 °C. The prepared films were stored in a dark, ventilated space in a container with moisture-absorbing silica beads.

### 2.4. Characterization Methods

The Fourier-transform infrared (FTIR)-spectra of the films were collected with PerkinElmer Spectrum One (Waltham, MA, USA) using an attenuated total reflection (ATR) setup. The measurement was made at room temperature from a circular piece cut from the initial material, taking four scans with a range of 500–4000 cm^−1^.

The thermogravimetric analysis (TGA) measurements of the films were made with Mettler-Toledo TGA850 (Columbus, OH, USA). The samples (8–13 mg) were placed in alumina crucibles and heated from 30 to 700 °C at 10 °C/min after an isothermal step at 30 °C for 10 min.

Differential scanning calorimetry (DSC) measurements to determine the glass- (*T*_g_) and phase-transition temperatures were made with TA-Instruments DSC Q2000 (New Castle, DE, USA). For the *T*_g_ measurements, the samples dried under ambient conditions were placed in aluminum pans, equilibrated at −65 °C, heated from −65 to 150 °C at 20 °C/min to remove the thermal history, cooled back from 150 to −65 °C at 20 °C/min, and heated again from −65 to 150 °C at 20 °C/min. The *T*_g_ was determined from the midpoint of the second heating curve (Figure S3, Supplementary Materials). To study the stimuli-response of the films, the samples equilibrated overnight in carbonate buffer at a pH of 10 were plotted dry and placed into hermetically-sealed aluminum pans. The samples were equilibrated to −50 °C, heated from −50 to 100 °C at 10 °C/min, cooled back to −50 °C at 10 °C/min, and finally heated from −50 to 100 °C. The second heating runs were analyzed. The peak temperature and enthalpy of the transitions were determined by fitting a linear integral to the curve from 10 to 100 °C (Figure S5, Supplementary Materials) in Universal Analysis software from TA-Instruments.

The mechanical analyses of the films were made with TA-Instruments Q800 DMTA (New Castle, DE, USA). Stress–strain measurements were made at room temperature from rectangular samples by ramping up the stress with 3 N/min.

The swelling capacity of the films was determined from uniformly shaped samples by immersing the samples into deionized water for 24 h. The wet mass was determined by carefully drying the surface by blotting and measuring the wet mass (*m*_wet_). The dry mass (*m*_dry_) was determined after drying the samples for 24 h in a vacuum desiccator. 

The swelling capacity (SW) was calculated as follows:

$$SW=\frac{m_{wet}-m_{dry}}{m_{wet}}\times 100$$

The transmittance of the films was measured with a Jasco V-750 UV spectrophotometer (Easton, MD, USA) with a Jasco CTU-100 temperature control unit (Easton, MD, USA) using 10 mm quartz cuvettes. The samples were equilibrated in a pH 10 carbonate buffer at least 12 h before measurement, attached to a plastic holder with tape, and immersed in a pH 10 carbonate buffer in standard quartz cuvettes. The transmittance was set to 100% after equilibration at room temperature. The transmittance changes were monitored upon heating twice from 20 to 80 °C at 10 °C/min. The second heating run was analyzed.

Scanning electron microscopy (SEM) measurements were carried out using a Hitachi S-4800 FESEM (Tokyo, Japan). The film samples were cut by hand using a scalpel, placed on a SEM stand with two-sided carbon tape, and dusted with pressurized air.

The transmission electron microscopy (TEM) measurements were carried out using a Jeol JEM-1400 transmission electron microscope (Tokyo, Japan). Thin cross-sectioned film samples were prepared by a Leica EM Ultracut UC6i ultramicrotome (Wetzlar, Germany).

## 3. Results and Discussion

### 3.1. Film Preparation

**Scheme 1:**
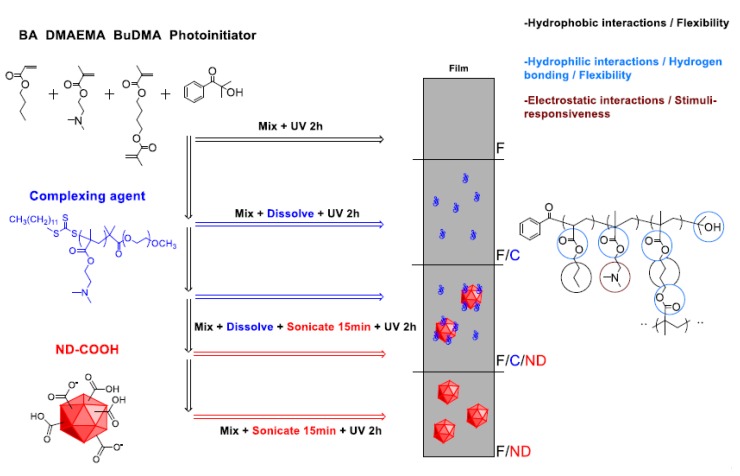
Schematic representation of film preparation. **Black**: Addition of monomers and mixing. **Blue**: Addition of a complexing agent and dissolving. **Red**: Addition of nanodiamonds (NDs) and sonication.

Composite films with a thickness of around 200 um were made by the photopolymerization of BA and DMAEMA in the presence of 2.5 wt % of the cross-linker BuDMA. In order to study the effects of the NDs and the PDMAEMA-*b*-PEO complexing agent, a series of films were prepared by varying the ND content and the use of the block copolymer complexing agent (Table 1). The NDs and PDMAEMA-*b*-PEO were added either separately or at the same time (Scheme 1).

**Figure 1:**
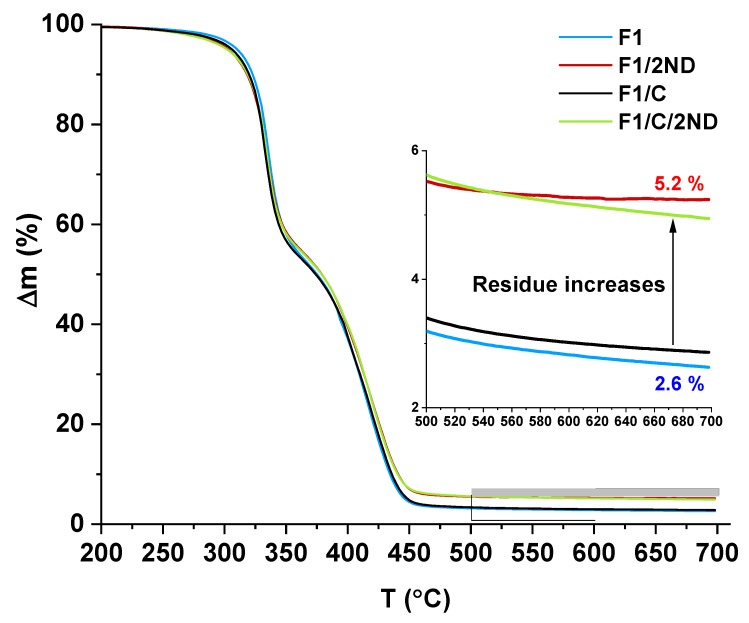
Thermogravimetric analysis (TGA)-curves of films with different filler contents.

The IR-spectra and a single *T_g_* transition in the DSC runs confirmed the copolymerization of the monomers (Figures S1, S3, and S4, Supplementary Materials). The TGA-curves of the films contained two distinct steps corresponding to PDMAEMA and poly(butyl acrylate) (PBA). The first was assigned to PDMAEMA and the second to PBA. According to the measurements made with increasing the BA content (Figure S2, Supplementary Materials). The addition of NDs or NDs with the complexing agent did not considerably change the thermal decomposition profile, but resulted in an increase of the residue after the heating (Figure 1). The increase of the residue corresponded to the amount of ND added to the polymer mixtures before curing.

### 3.2. Film Properties and the Effects of the Fillers 

Pure PBA and PDMAEMA polymers have very different glass transition temperatures—PBA −53 °C and PDMAEMA 12 °C [37]. For the copolymerized films in the present case, a single *T*_g_ was seen for all of the samples (Table 2 and Figure S4, Supplementary Materials). The observed *T*_g_ values were a few degrees higher than what was theoretically expected using the Fox equation for linear copolymers with similar compositions. The higher value of *T*_g_ demonstrated the effect of the cross-linking, and was essentially the same as that found earlier for the corresponding copolymer films [26]. The *T*_g_ of the films with added NDs and/or a complexing agent showed only marginal differences when compared to the films without fillers (Figure S4 and Table 2).

**Figure 2:**
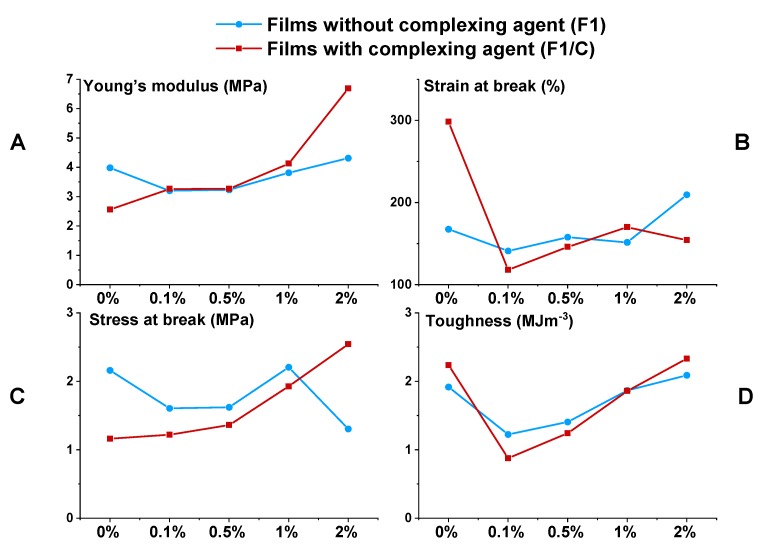
Mechanical properties of films with different ND filler contents. (**A**) Young’s modulus, (**B**) strain at break, (**C**) stress at break, and (**D**) toughness.

**Figure 3:**
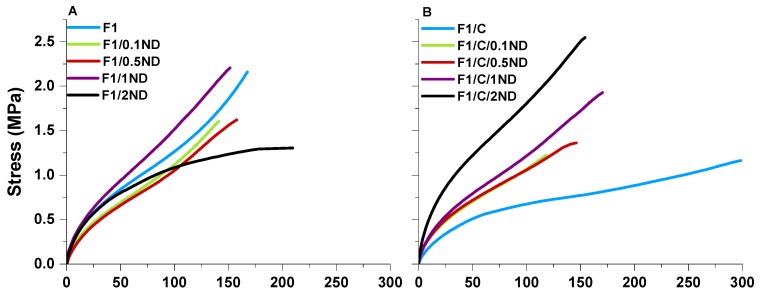
Stress–strain curves of films with an (**A**) ND filler and with an (**B**) ND and complexing agent.

The mechanical analysis of the films without a complexing agent (F1/ND) revealed that the addition of NDs of up to 2 wt % had overall a small effect on their mechanical properties (Figure 2 and Figure 3, and Table 2). The addition of NDs in amounts higher than 2 wt % led to sedimentation, and increasing the amount further was not attempted. Thus, the unaltered mechanical properties are connected with the aggregation and sedimentation of NDs, which has been earlier observed in ND/epoxy composites [18].

When the block copolymer complexing agent was added without NDs, it resulted in clearly softer films (F1/C) when compared with the films without block copolymer (F1; Figure 2 and Figure 3). The plasticizing effect was due to the added linear block copolymers that reduced the overall crosslink density of the material. When NDs were added together with the complexing agent, the change from the base material (F1/C) was obvious, right from the smallest filler content of 0.1 wt %. The most obvious change was the decrease in strain at the break when the NDs were added, indicating that the NDs formed physical crosslinks with the matrix and the block copolymer chains, stiffening the network, but leading to failure at the lower strains. Upon further addition of the NDs, the mechanical properties built up with increasing ND content, and the combination of the NDs and the complexing agent yielded, at best, a material F1/C/2ND that had over a ~161% increase in modulus and ~118% increase in stress at break. We rationalized that this was due to the dispersing effect of the PDMAEMA-b-PEO on the NDs, combined with the block copolymer becoming a part of the network during polymerization. The dispersing effect was important during the film preparation, as the added block copolymer provided stability to the filler dispersion, and simultaneously increased the viscosity of the liquid medium.

**Figure 4:**
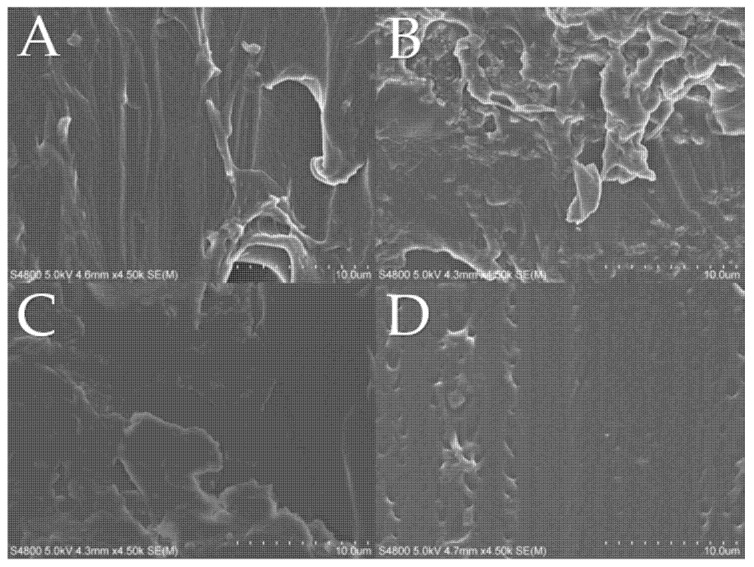
Scanning electron microscopy (SEM) cross-section images of films (**A**) F1, (**B**) F1/2ND, (**C**) F1/C, and (**D**) F1/C/2ND. Scale bar = 10 um.

**Figure 5:**
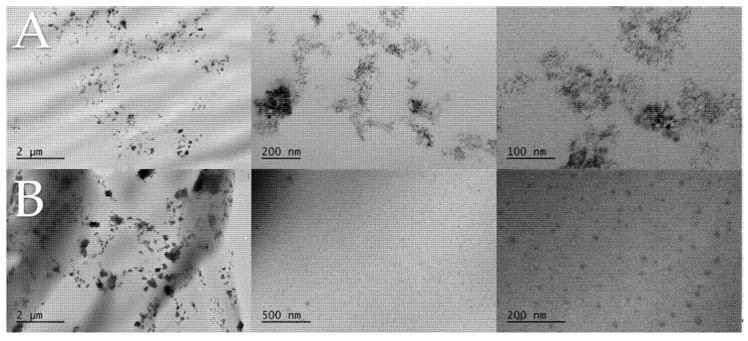
Transmission electron microscopy (TEM) cross-section images of films (**A**) F1/2ND and (**B**) F1/C/2ND. Magnification from left to right.

The dispersion of the NDs into the polymer matrix was confirmed by studying the distribution of the NDs within the films by SEM and TEM (Figure 4 and Figure 5). When comparing the films, aggregates were observed in F1/2ND that could not be seen in the F1/C/2ND or the films without NDs. However, a closer look at the cross-sections with TEM revealed similar-sized clusters in both F1/2ND and F1/C/2ND (Figure 5). Areas of very well dispersed NDs, which were observed in F1/C/2ND, were absent in F1/2ND (Figure 5B). This indicated that NDs were more evenly distributed in the matrix with the help of the complexing agent.

### 3.3. Swelling Capacity and Stimuli-Responsive Behaviour of the Films

**Figure 6:**
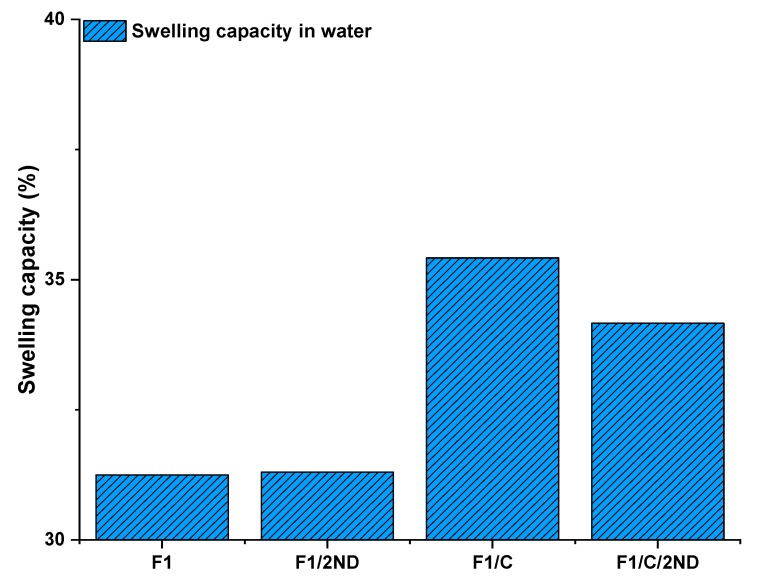
Swelling capacity of F1, F1/2ND, F1/C, and F1/C/2ND in water.

When comparing the swelling properties in water, slight increases in swelling were observed for the ND-containing F1 films (Figure 6 and Figure S10, Supplementary Materials). When the block copolymer was present, the water uptake increased because of the hydrophilic chains, and when the NDs were added, the swelling decreased. In water, complex formations between negatively charged NDs and positively charged DMAEMA amino-groups took place [36] in pHs between the pKas of the basic (pKa of DMAEMA = ~7.5 [38]) and acidic (pKa of COOH = ~4–5 [39]) units. The results showed that in the case of dispersed NDs (Figure 5), they restricted the swelling of the films, likely because of the electrostatic interactions between the NDs and DMAEMA units. 

**Figure 7:**
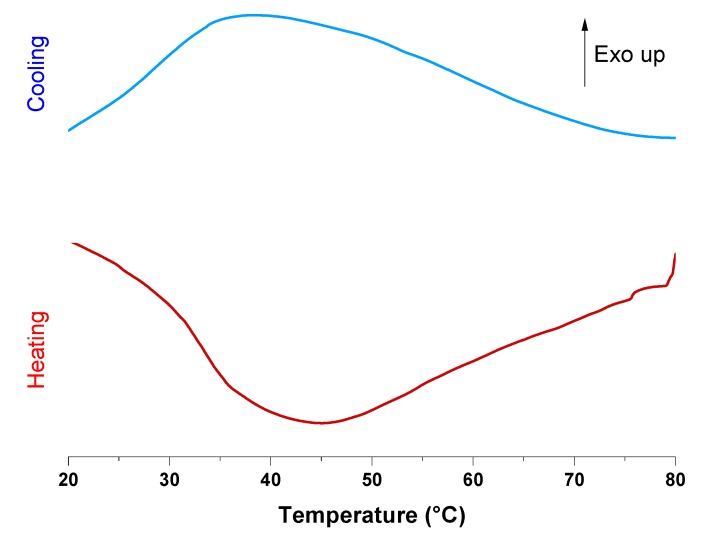
Reversible phase-transition of the films observed in the differential scanning calorimetry (DSC) runs (sample F1).

Aqueous PDMAEMA solutions are known to be thermoresponsive when sufficient amounts of the repeating units are non-protonated, in other words, above the pKa of PDMAEMA [40]. In the calorimetric measurements of the water-swollen films, no enthalpy changes were observed upon the heating and cooling scanning. However, for the films equilibrated in the pH 10 carbonate buffer, a broad, reversible phase-transition was observed upon heating and cooling for all films, which showed that the copolymerized DMAEMA units also participated in the phase transition process (Figure 7, Figures S6 and S7, Supplementary Materials). The peak temperatures of the phase transitions were around 45 °C for all of the films (Figures S6 and S7, Supplementary Materials), The enthalpy of transitions were between 10–15 J/g, which corresponded to the 2–3 kJ/mol per repeating unit of DMAEMA. These values were well in line with the enthalpies reported for linear PDMAEMA solutions at a pH of 8–9 [40].

**Figure 8:**
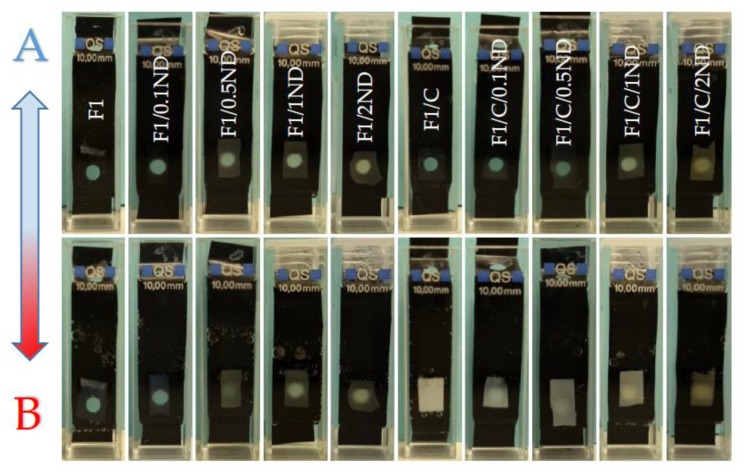
Phase transition of the films visualized as follows: (**A**) room temperature samples and (**B**) heated samples. Heat-cool-heat cycle (Figure S9, Supplementary Materials).

**Figure 9:**
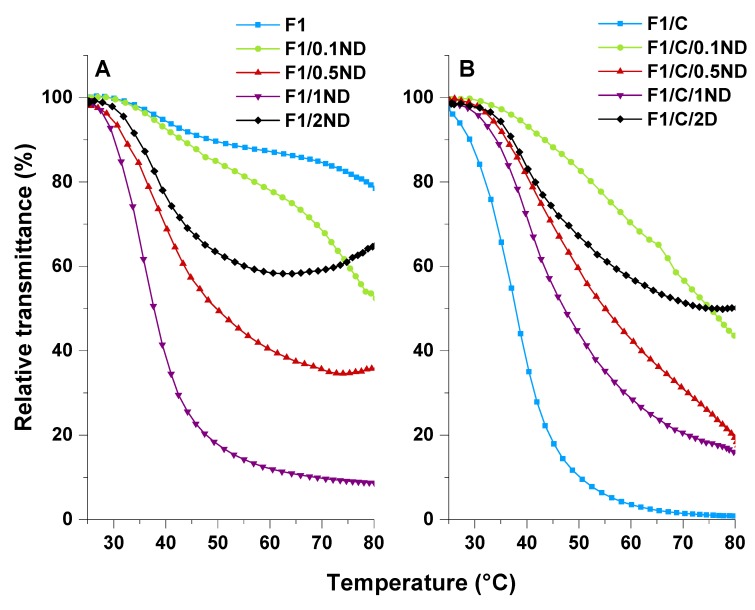
Relative transmittance of the (**A**) F1 films and the (**B**) F1/C films immersed in a pH 10 buffer.

The phase transition was also clearly demonstrated as a change in transmittance upon heating (Figure 8 and Video S1, Supplementary Materials). At the same time, the films contracted slightly. The changes were reversible, as suggested by the DSC measurements. To gain more insight into the effects of the fillers, the UV transmittance of the films was measured and the changes were monitored (Figure 8 and Figure 9). It should be noted that the changes in the film transmittance were measured relative to the starting value, and that the ND-filled films were more opaque in the beginning. For the F1 film, a gradual loss of transmittance was observed upon heating, starting from 35 °C. Film F1/C, on the other hand, showed an abrupt phase-transition already starting from 25 °C, and resulted in a completely opaque film above 70 °C. This response clearly stemmed from the PDMAEMA-*b*-PEO block copolymer chains, which, upon the heating phase, separated more effectively compared with the F1 film.

The addition of NDs made the films increasingly more opaque, even at room temperature (Figure 8). Without the block copolymer, the addition of NDs lowered the onset temperature of the phase-transition and sharpened the transmittance change compared with the pure F1 film (Figure 9). For the films with a block copolymer, the effect was the opposite—the onset of the transmittance change was shifted to higher temperatures than the pure F1/C film. Otherwise, the behavior was the same when more NDs were added. We hypothesize that an increase in ND content increases thermal conduction, lowering the onset through more effective heat transfer through the material. At the same time, NDs bind to the matrix, as shown in the changes of the mechanical properties, making the phase separations at higher ND contents slower. This explains the changes in the onset temperatures in the presence of a block copolymer when NDs are added, as the previously free block copolymer is bound with the NDs, and is thus unable to phase separate as effectively. However, the onset, sharpness, and the extent of the transmittance changes, did not change linearly with the ND content in either film series. It is clear that the optical properties originated from a combination of the distribution of the NDs, ND–polymer interactions, and the phase transition phenomena in the matrix.

## 4. Conclusions

A series of cross-linked, 0–2 wt % nanodiamond (ND)-containing poly(butyl acrylate-*co*-dimethylaminoethyl methacrylate) (P(BA-*co*-DMAEMA)) composite films were prepared by photopolymerization with or without a linear block copolymer complexing agent, PEO-*b*-PDMAEMA. The addition of the carboxylate-functionalized NDs to P(BA-*co*-DMAEMA) films up to 2 wt % only slightly changed the mechanical properties as a result of the aggregation and sedimentation of the NDs. When the complexing agent PEO-*b*-PDMAEMA was used, it improved the ND dispersion and interaction with the P(BA-*co*-DMAEMA) matrix, as the mechanical properties changed at already low ND contents and led to a 161% increase in Young’s modulus and a 118% increase in stress at break for the highest studied ND filling. 

Apart from the mechanical properties in the dry state, the water uptake and stimuli-responsivity of the films were studied. The addition of NDs did not markedly change the swelling of the P(BA-*co*-DMAEMA) composites. The films prepared with the complexing agent had a slightly higher water uptake, which was suppressed upon the addition of 2 wt % NDs, because of the complexation between negatively charged NDs and cationic DMAEMA units. The films equilibrated in a pH 10 buffer showed a reversible thermal phase-transition originating from the PDMAEMA. The effect of the ND addition or the use of the complexing agent did not change the calorimetrically observed phase-transition temperature or the enthalpy of the transitions, but the transmittance change shifted to lower temperatures upon the addition of NDs. The change in optical transmittance was significantly amplified by the introduction of the PEO-*b*-PDMAEMA block copolymer.

## Figures and Tables

**Table 1:** Film compositions. BA—butyl acrylate; DMAEMA—dimethylaminoethyl methacrylate; ND—nanodiamond; PDMAEMA-*b*-PEO—poly(dimethylaminoethyl methacrylate)-*block*-poly(ethylene oxide).

Film Label	BA wt %	DMAEMA wt %	ND wt %	PDMAEMA-*b*-PEO wt %
F1	20	80	0	0
F1/0.1ND	20	80	0.1	0
F1/0.5ND	20	80	0.5	0
F1/1ND	20	80	1	0
F1/2ND	20	80	2	0
F1/C	20	77.5	0	2.5
F1/C/0.1ND	20	77.5	0.1	2.5
F1/C/0.5ND	20	77.5	0.5	2.5
F1/C/1ND	20	77.5	1	2.5
F1/C/2ND	20	77.5	2	2.5

**Table 2:** Mechanical properties of the films.

Film Label	*T_g_* (°C)	Young’s Modulus (MPa)	Strain at Break (%)	Stress at Break (MPa)	Toughness ^1^ (MJ·m^−3^)
F1	5.0	3.9	167.6	2.2	1.9
F1/0.1ND	6.1	3.2	141.1	1.6	1.2
F1/0.5ND	9.1	3.2	157.8	1.6	1.4
F1/1ND	5.9	3.8	151.5	2.2	1.9
F1/2ND	5.0	4.3	209.5	1.3	2.1
F1/C	7.0	2.6	298.5	1.2	2.2
F1/C/0.1ND	7.2	3.3	118.1	1.2	0.9
F1/C/0.5ND	6.6	3.3	146.0	1.4	1.2
F1/C/1ND	7.3	4.1	170.2	1.9	1.9
F1/C/2ND	7.3	6.7	154.0	2.5	2.3

^1^ Calculated by integrating the stress–strain curve.

## Supplementary Materials

The following are available online at https://www.mdpi.com/2073-4360/12/3/507/s1: Figure S1: IR-Spectra of the prepared film F1; Figure S2: TGA with increasing BA content showing that PDMAEMA degrades the first and BA second dry sample; Figure S3: Figure S3. Determination of Tg from the DSC curve with effect of BA content; Figure S4: Glass transition of films; Figure S5: Determination of phase transition properties from DSC; Figure S6: DSC-curves of the phase transition of films without complexing agent; Figure S7: DSC-curves of the phase transition of films with a complexing agent; Figure S8: Film fabrication equipment; Figure S9: Heat (A), cool (B), heat (C) cycle of F1/C (Video S1); Figure S10: Swelling capacities of all films; Table S1: Film recipe; Video S1: Thermoresponsivity.
