# Supplementary material for: Stimuli-Responsive Nanodiamond–Polyelectrolyte Composite Films
Source: Polymers (Basel). 2020 Feb 26;12(3):507. doi: 10.3390/polym12030507 (PMC7182812; doi:10.3390/polym12030507)
Supplement: Supplementary file 1 [file polymers-12-00507-s001.zip › polymers-720218/Supplementary-polymers-720218.pdf]

Stimuli-Responsive Nanodiamond-Polyelectrolyte Composite Films

Supporting information

Tony Tiainen, Marina Lobanova, Erno Karjalainen, Heikki Tenhu, Sami Hietala

Department of Chemistry, University of Helsinki, PB 55. FIN-00014 HU

Corresponding author* Sami.Hietala@helsinki.fi

Film recipes

Table S1. Film recipe.

**Reagent**	**wt%****	**M (g/mol)**	**D (g/ml)**
BA	20	128.17	0.890
DMAEMA	80	157.21	0.933
BuDMA (Crosslinker)	2.5	226.27	1.023
2-Hydroxy-2-methylpropiophenone (Initiator)	1.0	163.2	1.077
PDMAEMA-PEO (Complexing agent)*	2.5		
ND*	0.1 - 2.0		

*If added.
**The main precursor components (BA, DMAEMA, PDMAEMA-b-PEO) add up to 100 wt%. wt% of additives is calculated from the total mass of main precursor components. For comparison, films with 70- and 80 wt% BA were prepared.

IR

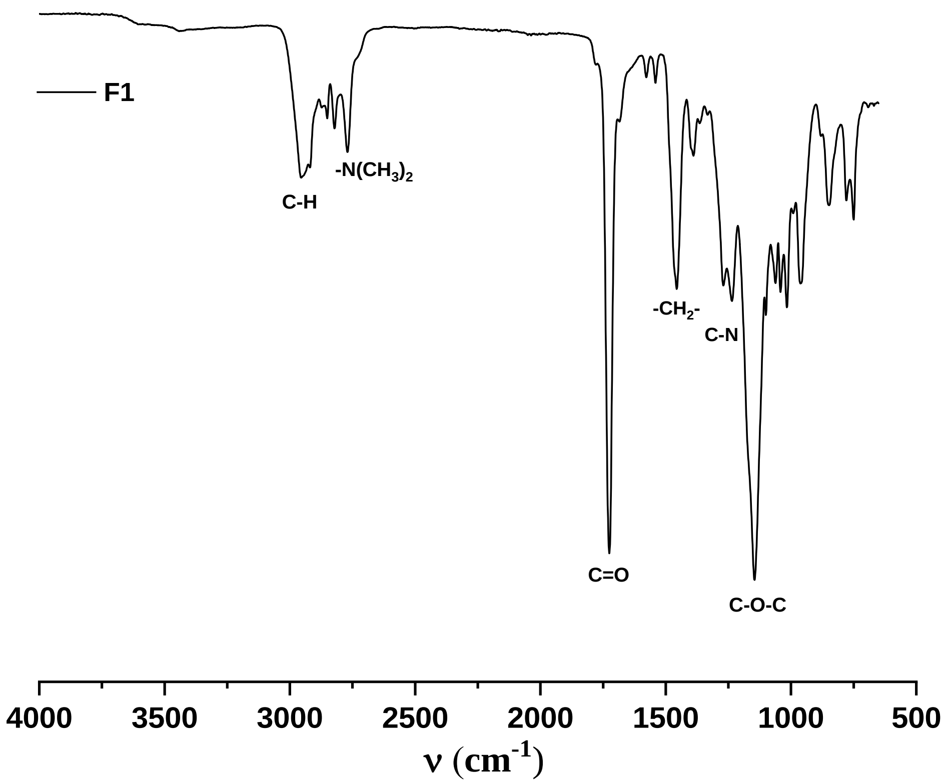

Figure S1. IR-Spectra of prepared film F1.

TGA

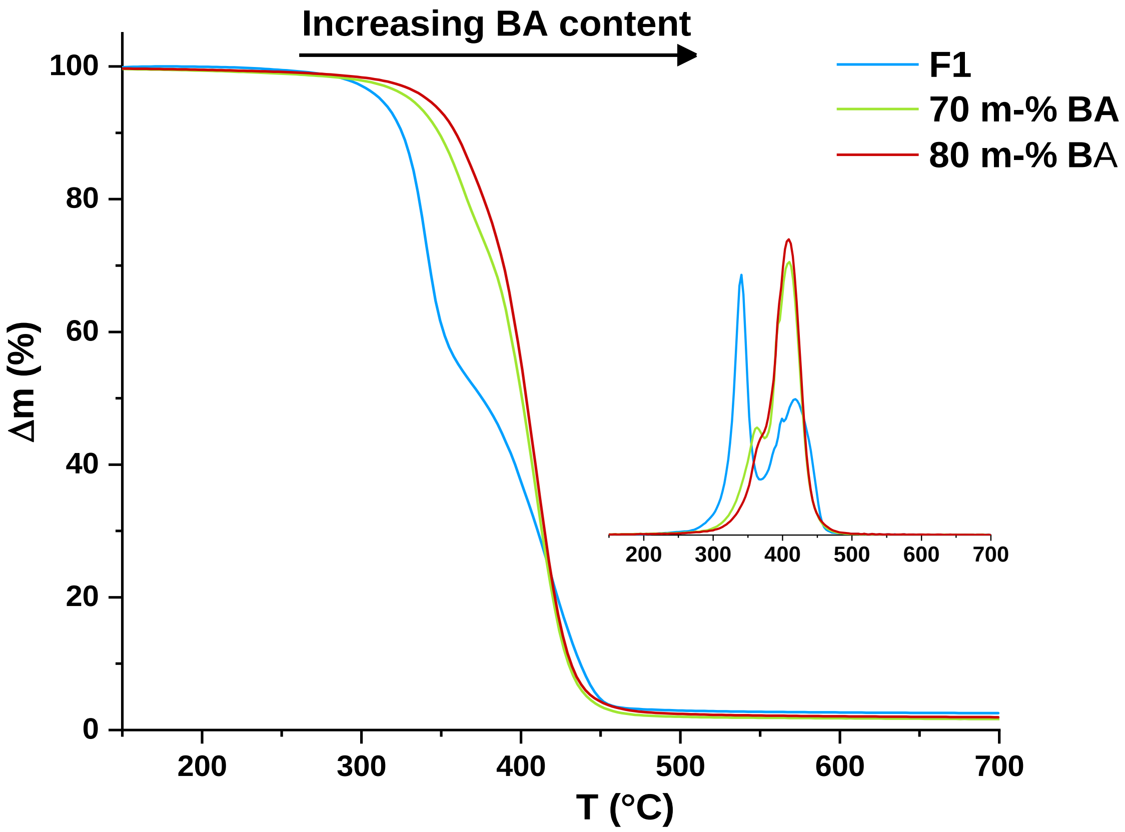

Figure S2. TGA with increasing BA content showing that PDMAEMA degrades first and BA second. Dry sample.

DSC

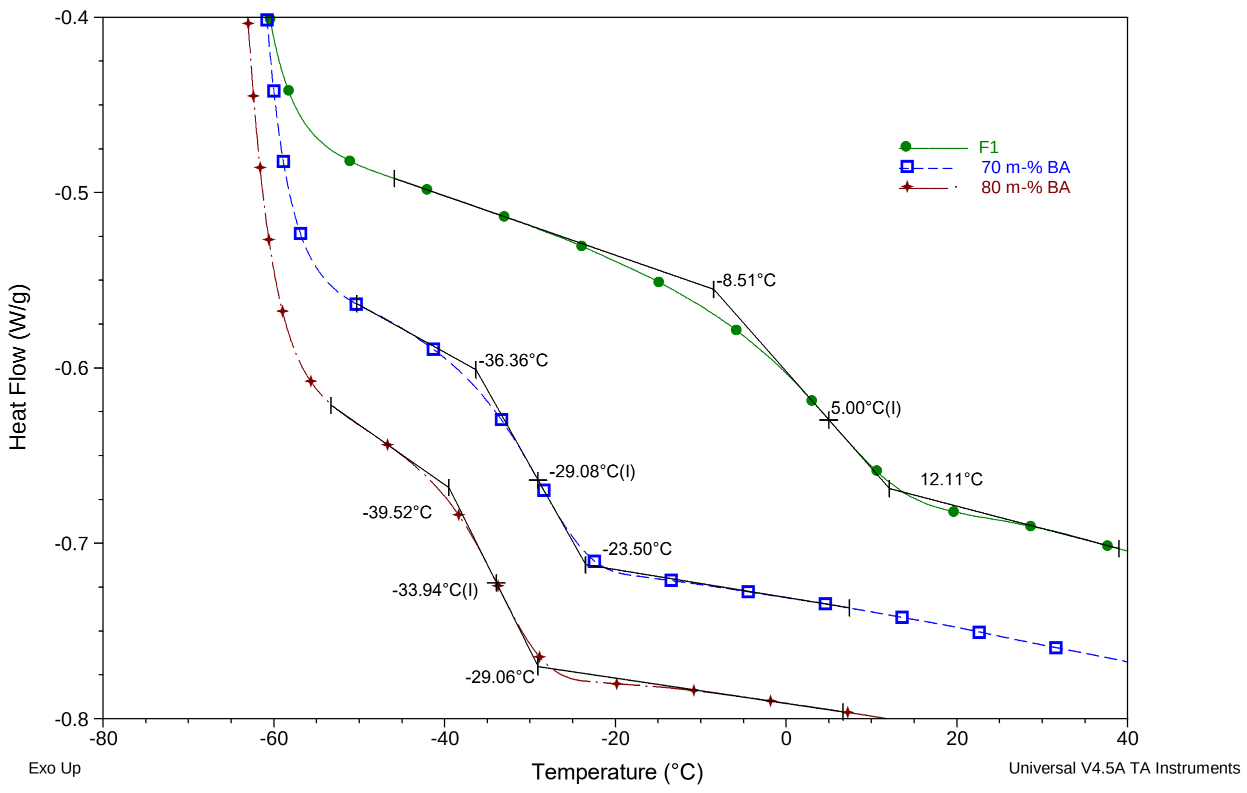

Figure S3. Determination of T_g from the DSC curve with effect of BA content.

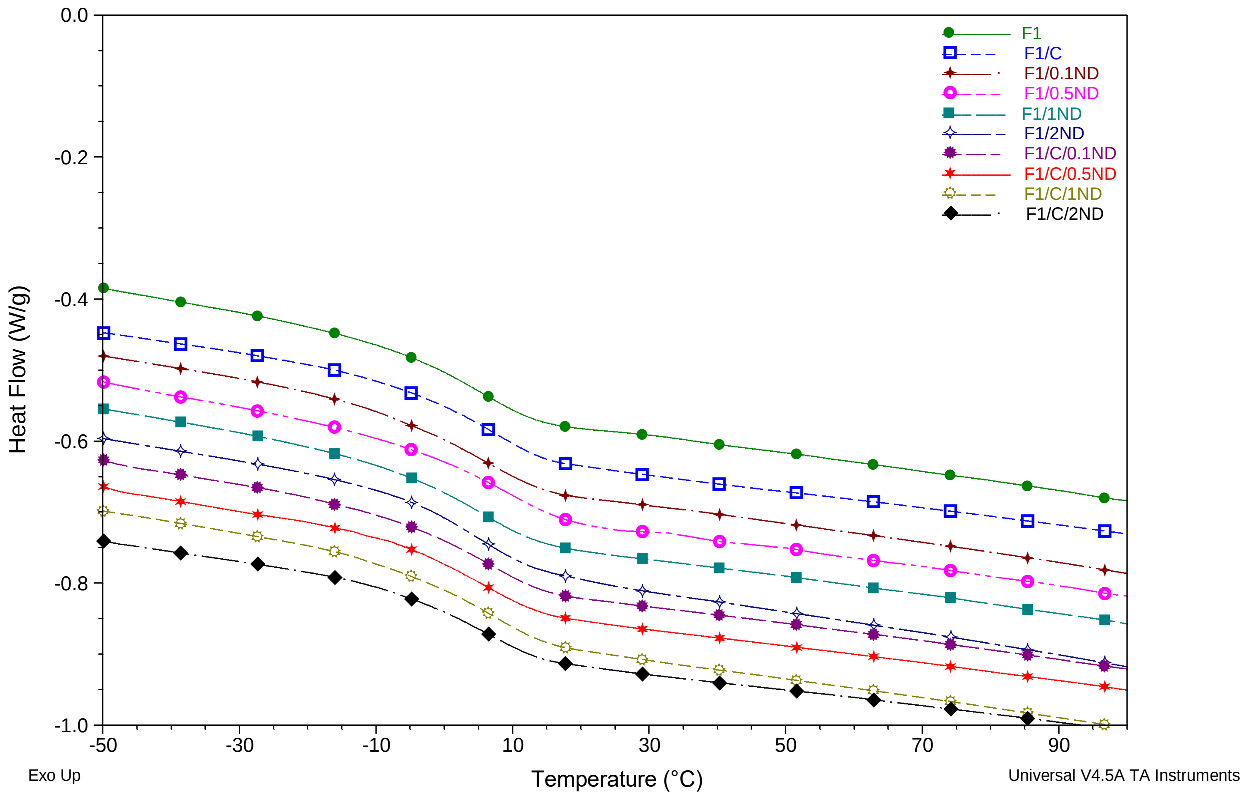

Figure S4. Glass transitions of films.

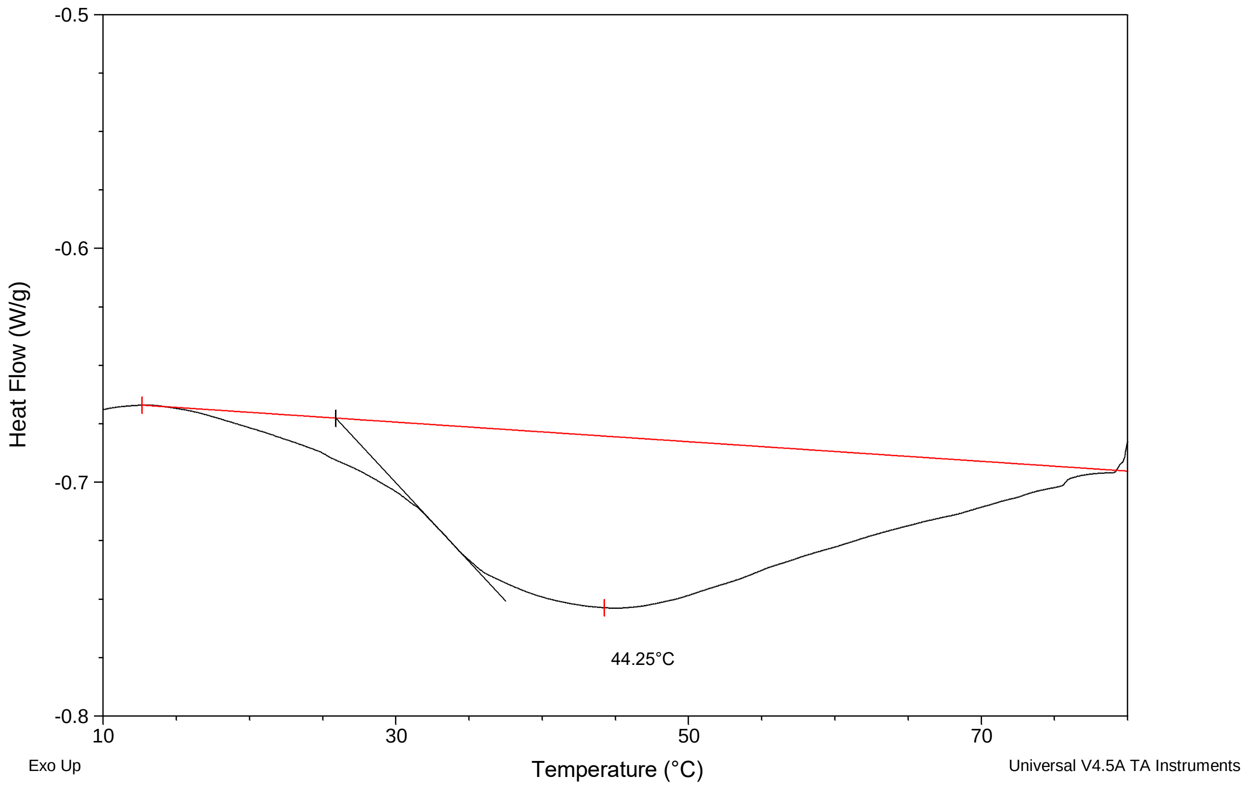

Figure S5. Determination of phase transition properties from DSC.

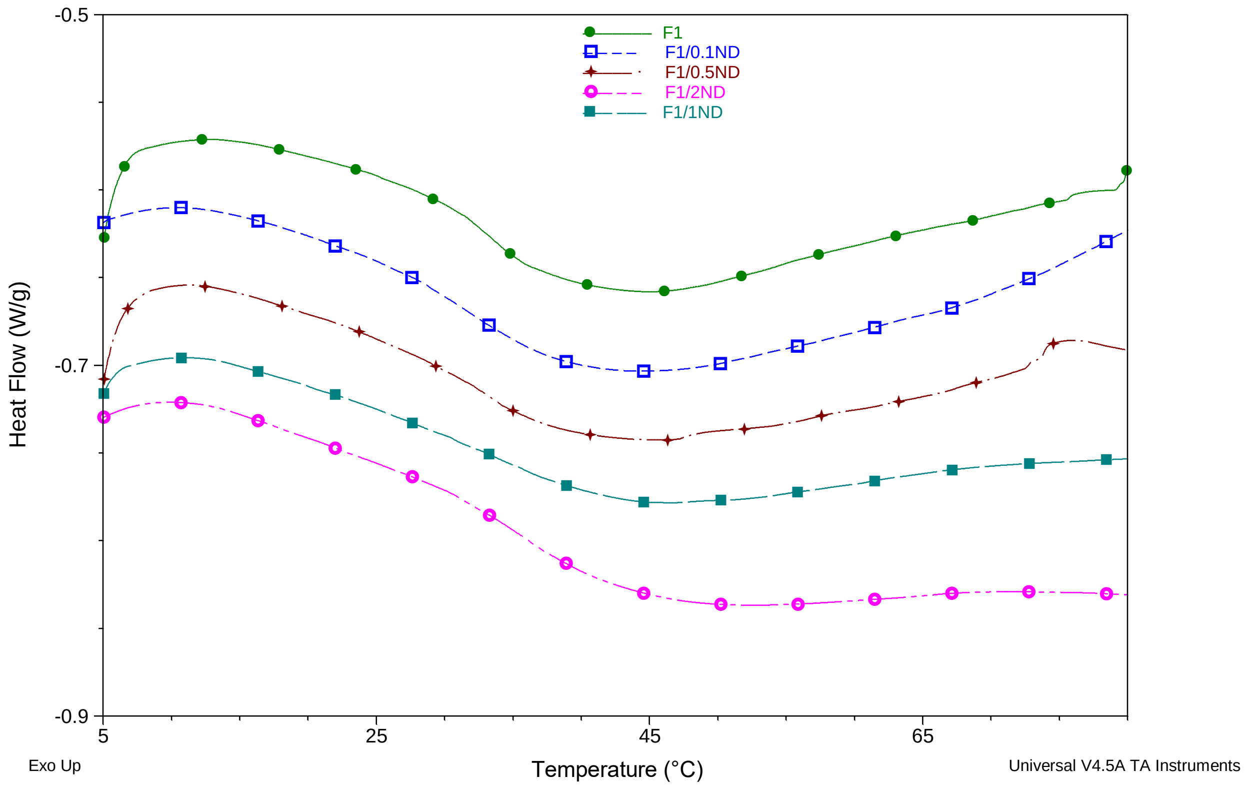

Figure S6. DSC-curves of the phase transition of films without complexing agent.

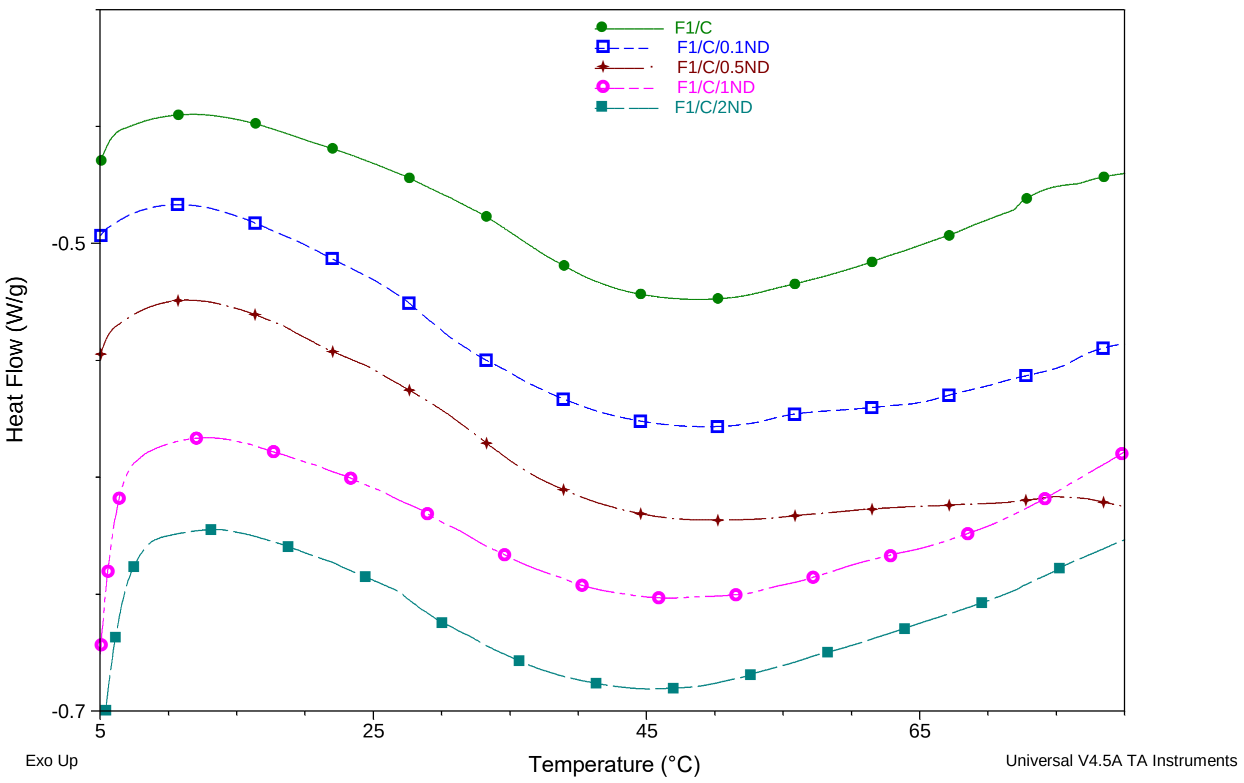

Figure S7. DSC-curves of the phase transition of films with complexing agent.

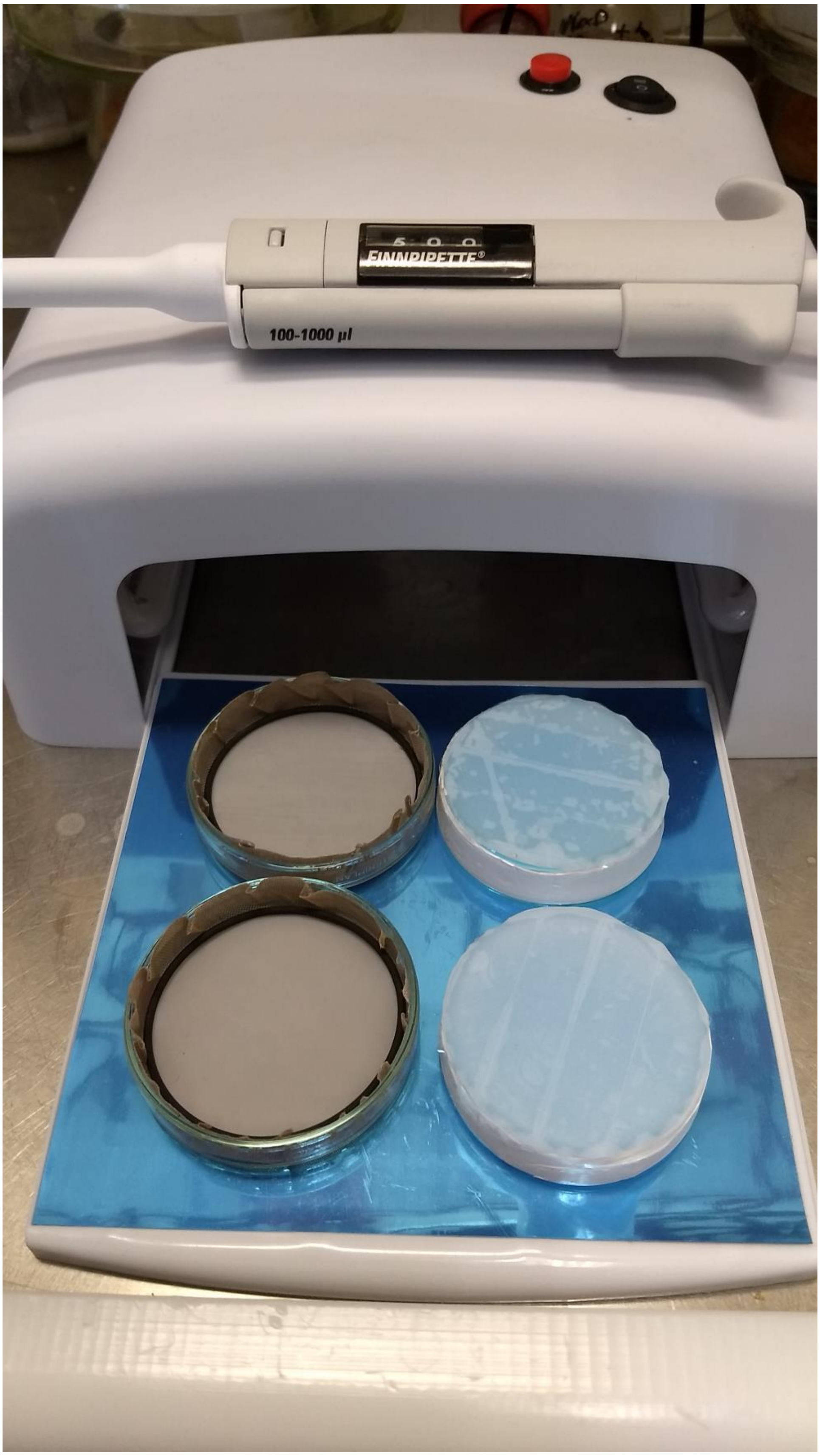

Figure S8. Film fabrication equipment.

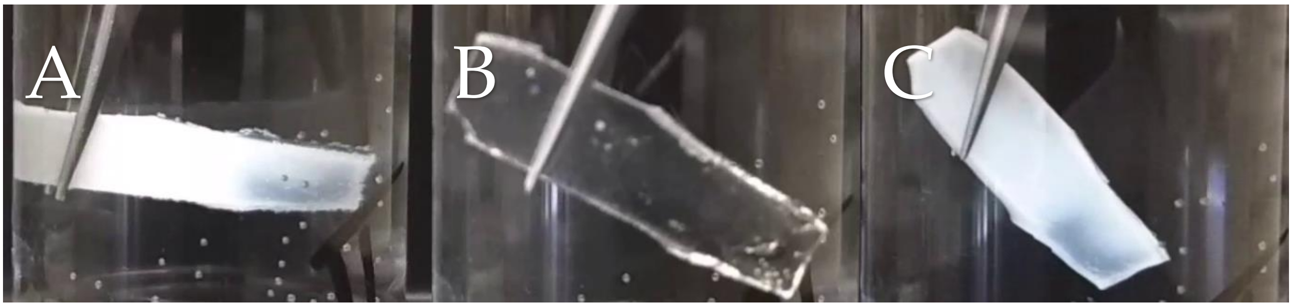

Figure S9: Heat (A), cool (B), heat (C) cycle of F1/C (Video S1).

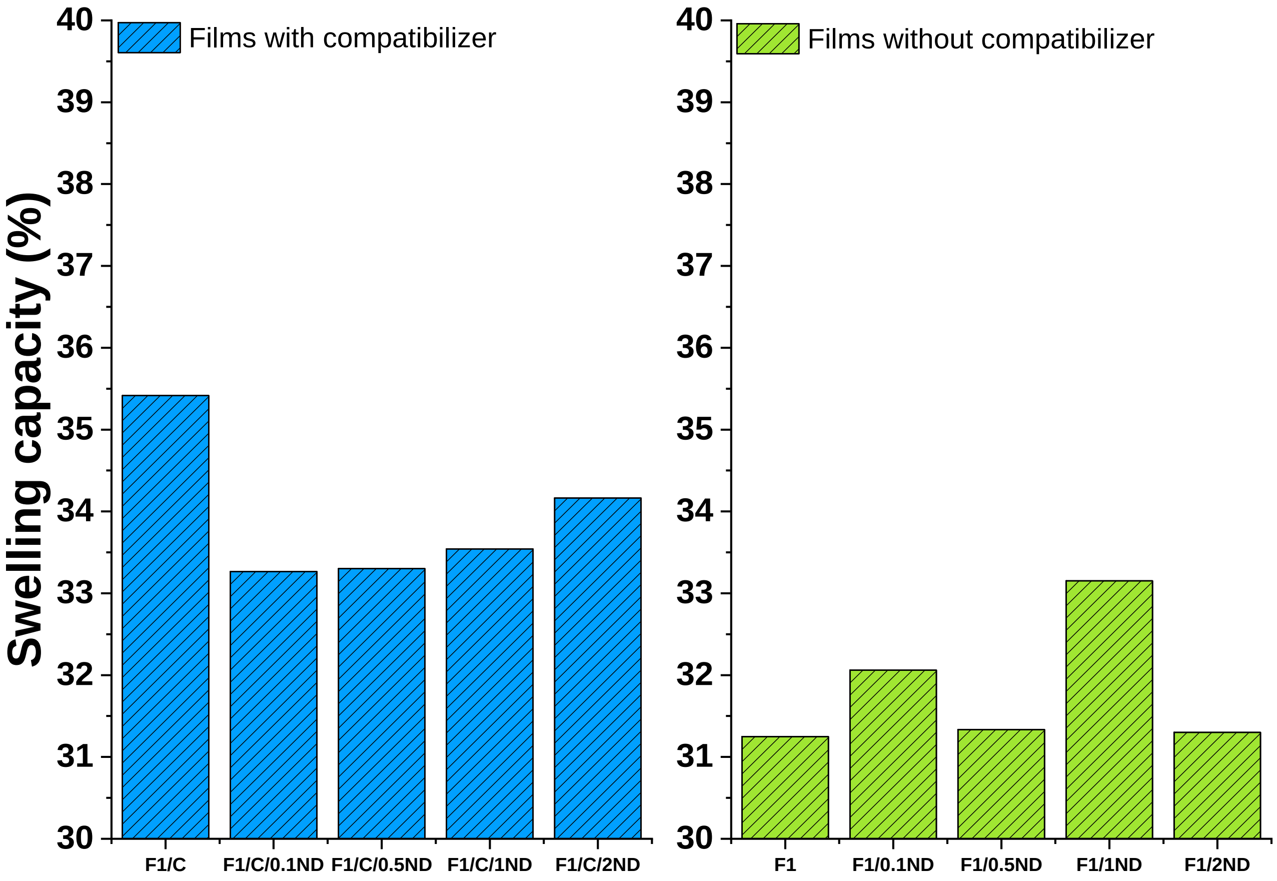

Figure S10. Swelling capacities of all films.
